# Hodgkin’s Lymphoma as an Unusual Cause of Eosinophilic Pleural Effusion

**DOI:** 10.7759/cureus.16994

**Published:** 2021-08-08

**Authors:** Husain Kadhem, Naser Naser, Sayed Mohammed Jawad Alwedaie, Sayed Ali Hashem Neama, Rabab Alkhayyat

**Affiliations:** 1 Internal Medicine/Respiratory Unit, Salmaniya Medical Complex, Manama, BHR; 2 Pathology and Laboratory Medicine, Salmaniya Medical Complex, Manama, BHR

**Keywords:** pulmonary medicine, hodgkin’s lymphoma, eosinophilic pleural effusion, radiology, histopathology

## Abstract

Eosinophilic pleural effusion can be the first presenting feature of a wide range of diseases, with malignancy being the commonest cause. Elevated levels of eosinophils could be an indicator of a favourable prognosis. In clinical practice, malignant lymphomas have been rarely associated with eosinophilic pleural effusions. In this report, we present the case of a 37-year-old otherwise healthy woman, who initially presented with a cough of five months' duration. Diagnostic workups including pleural and lymph node biopsies confirmed the diagnosis of nodular lymphocyte-predominant Hodgkin’s lymphoma.

## Introduction

Eosinophilic pleural effusion, which was first described by Harmsen in 1894, is explained to be a pleural effusion that consists of at least 10% eosinophils among the total leukocyte’s count. Although previously it was thought that the presence of eosinophils denotes a benign pathology [1], recent studies have come to a different conclusion by digging deeper in this field. Here, we present a rare case of eosinophilic pleural effusion caused by a specific histological type of Hodgkin’s lymphoma. To our best knowledge, eosinophilic pleural effusion is rarely associated with Hodgkin’s lymphoma, with very few available case reports [2,3]. 

## Case presentation

**Figure 1:**
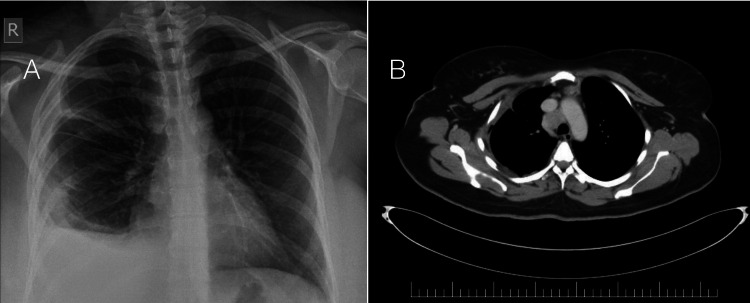
Patient's chest X-ray and computerised tomography Chest X-ray (image A) demonstrates obliterated right costophrenic angle, suggestive of pleural effusion. Computerised tomography (image B) of the chest revealed a large right paratracheal lymph node measured at 2.6 × 2.6 × 3.2 cm with central low attenuation suggestive of necrosis. R: right side.

A 37-year-old female patient, not a known case of any medical illness, presented with a five-month history of cough. It was not associated with sputum production, shortness of breath, fever, night sweats, appetite change or weight loss. Prior to this presentation, the patient had visited several hospitals where chest X-ray and some laboratory tests were ordered. She received three courses of antibiotics without any significant improvement in her condition. Upon physical examination, the patient was well built and not distressed. Upon close examination of the chest, no skin lesions, mass or swellings were observed or felt. However, on auscultation, diminished breath sounds on the right lower lung zones were noticed. A new chest X-ray was ordered, which showed obliteration of right costophrenic angle, suggestive of pleural effusion (Figure 1A).

**Figure 2:**
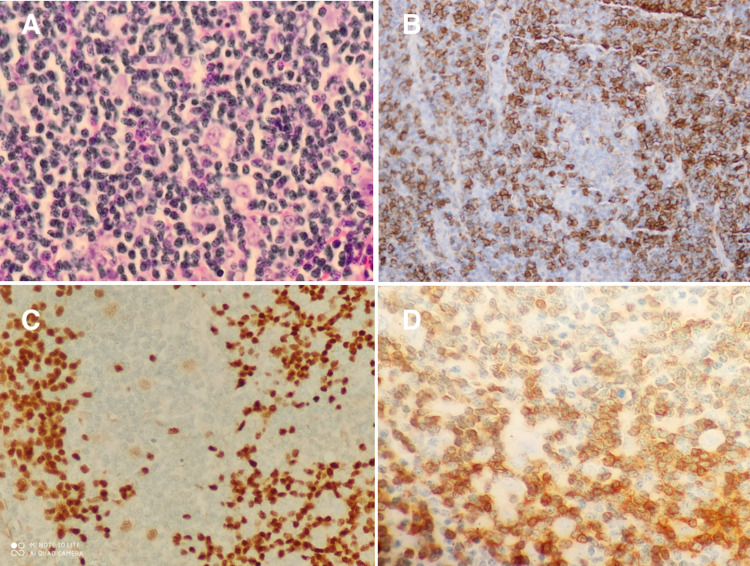
Histopathological study Image A (hematoxylin and eosin) shows a histological slide of the right paratracheal lymph node. This image illustrates effaced architecture with a nodular pattern. There are large nodules composed mostly of small lymphocytes with occasional large atypical lymphocytes. The atypical lymphocytes show bi- to multilobed nuclei and distinct nucleoli. Occasional small granulomas are noted in the background. No necrosis is noted. Images B, C and D show positive staining for CD20, OCT2 and CD3, respectively.

Subsequent chest ultrasound study revealed a moderate amount of pleural effusion. Sample pleural fluid was obtained using a pleural catheter device. The cell count of the obtained fluid showed white blood cells 2,450 cells/µL, red blood cells 70,000 cells/µL, neutrophils 13%, eosinophils 72%, lymphocytes 8%, proteins 0.048 g/L (serum protein was 76 g/L) and lactate dehydrogenase 316 U/L. The pleural fluid was considered to be exudative according to lactate dehydrogenase criteria for exudative pleural effusion. Pleural fluid culture and sensitivity test turned out to be sterile with negative acid-fast bacilli smear. Computerised tomography (CT) of the chest revealed multiple mediastinal lymph nodes, with the large one being the right paratracheal lymph node with central low attenuation, suggestive of necrosis (Figure 1B). No cavitation or bronchiectatic changes were appreciated in the lungs. No significant hilar or axillary lymph nodes were noticed according to the size criteria. A CT evaluation of the abdomen and pelvis was performed. Apart from minimal physiological fluids in the pouch of Douglas and a left-sided corpus luteal cyst, CT abdomen and pelvis revealed no significant finding. The patient underwent video-assisted thoracoscopy with pleural tissue and paratracheal lymph node biopsies. The pleural tissue biopsy demonstrated a gross appearance of congested fibrous tissue. Microscopically, the pleural tissue was infiltrated by chronic inflammatory infiltrate, fibrosis and congested blood vessels. The pathology report did not mention the presence of eosinophils within the pleural tissue. The right paratracheal lymph node biopsy was grossly fragmented and firm. Microscopically, the sections showed effaced architecture with nodular pattern (Figure 2). Like pleural tissue, no eosinophils were noticed in the lymph node. 

The final pathological study confirmed the diagnosis of nodular lymphocyte-predominant Hodgkin’s lymphoma. The patient was referred to haemato-oncology service for further assessments.

## Discussion

Eosinophilic pleural effusion (EPE) is defined as a pleural fluid in which its eosinophilic count comprises >10% of the white blood cells [4]. Previously, EPE was considered to be a predictor of benign pathology [1]. However, this belief was challenged by several studies [4-6], leading to a new understanding of this phenomenon.

A recent review study conducted by Li and his colleagues found malignancy to be the commonest cause of EPE [7], and its incidence has a close relationship with the population being studied and the diagnostic criteria being used. Among the causes of malignant EPE, lung cancer was found to be the leading cause with 23 reported cases, followed by non-Hodgkin lymphoma and metastatic carcinoma of unknown origin, each comprising 5% of total reported cases of malignant EPE [7]. Hodgkin's lymphoma is a rare cause of malignant EPE with only two reported cases [8,9].

An overwhelming number of recently published studies have demonstrated that the prevalence of malignancy could be as frequent in EPEs as in non-EPEs and that there is no statistically significant difference in the incidence of malignant neoplasm between the two [7].

With regard to clinical characteristics, patients with malignant pleural effusion tend to have a longer duration of symptoms than those with EPEs of benign pathologies [7]. The symptoms are usually cough, shortness of breath, chest discomfort, hemoptysis and weight loss, although asymptomatic presentations are also seen among a number of affected patients [7].

Most of the malignant EPE cases fail to respond to the available treatment options, and the current strategies are only used to provide symptomatic relief. These strategies include radiotherapy, chemotherapy, indwelling pleural catheter and thoracoscopy with pleurodesis [7]. With regard to disease prognosis, patients with malignant EPEs have a better prognosis than those with non-EPE malignancies, and in addition, higher eosinophilic count is shown to be associated with improved overall survival rates among patients with malignant EPE [7]. 

## Conclusions

Here, we discussed a rare case of eosinophilic pleural effusion caused by Hodgkin’s lymphoma. The current literature supports the idea that malignancies are the commonest cause of EPEs. However, haematological malignancies are rarely associated with EPE. The eosinophilic count has a tremendous prognostic significance for patients with malignant EPE, as a higher eosinophilic count is directly proportional to increased survival rates. The effectiveness of the current therapeutic strategies is yet to be confirmed by further research studies. 
